# Associations Between Screen Exposure in Early Life and Myopia amongst Chinese Preschoolers

**DOI:** 10.3390/ijerph17031056

**Published:** 2020-02-07

**Authors:** Gui-You Yang, Li-Hua Huang, Katrina L. Schmid, Chen-Guang Li, Jing-Yi Chen, Guan-Hao He, Li Liu, Zeng-Liang Ruan, Wei-Qing Chen

**Affiliations:** 1 Department of Biostatistics and Epidemiology, School of Public Health, Sun Yat-sen University, Guangzhou 510080, China; yanggy7@mail2.sysu.edu.cn (G.-Y.Y.); hlihua2@mail2.sysu.edu.cn (L.-H.H.); lichg3@mail2.sysu.edu.cn (C.-G.L.); chenjy246@mail2.sysu.edu.cn (J.-Y.C.); heguanh@mail2.sysu.edu.cn (G.-H.H.); liuli9931@163.com (L.L.); ruanzliang@mail2.sysu.edu.cn (Z.-L.R.); 2 School of Optometry and Vision Science, Faculty of Health, Queensland University of Technology, 60 Musk Ave, Kelvin Grove, Brisbane 4059, Australia; k.schmid@qut.edu.au; 3 Department of Information Management, Xinhua College of Sun Yat-sen University, Guangzhou 510080, China

**Keywords:** screen exposure, early life, myopia, preschoolers

## Abstract

This study aimed to explore the association between screen exposure in early life and preschool myopia. During the baseline survey of the Longhua Child Cohort Study (LCCS), data of 29,595 preschoolers were collected via a caregiver-reported questionnaire regarding children’s socio-demographic characteristics, visual status, screen exposure and relevant parental information. Data of 26,433 preschoolers with normal eyesight or myopia were included in the analysis and cox regression modelling was employed to assess the associations. Results suggested the hypothesis that screen exposure in early life could be significantly and positively associated with preschool myopia, and in agreement with this hypothesis was the association being strengthened with the increasing daily exposure duration and total years of exposure; in the stratification analysis based on the presence of parental myopia, these associations still existed, and the strength of associations was stronger in preschoolers with myopic parents than those without. Moreover, a statistically significant association was only observed between initial screen exposure that occurred during 0–1-years old and myopia for preschoolers without myopic parents, while the significant associations were observed between initial screen exposure that occurred during 0–1, 1–2, 2–3, and after 3 years old and myopia for preschoolers who had myopic parents, with the strongest association found in the group of children initially exposed to electronic screens during 0–1 year old. Thus our findings indicated the hypothesis that screen exposure in early life might be associated with the occurrence of preschool myopia, and that the postnatal first year might be the sensitive period for the association. However, it is premature to conclude that early screen time leads to myopia with current data. Further longitudinal studies performed with cycloplegia are necessary to verify the hypothesis and shed light on the more urgent question whether early screen exposure contributes to the later myopia epidemic of school-aged children.

## 1. Introduction

Myopia, most commonly defined as a spherical equivalent refraction (SER) of ≤−0.5D, is a risk factor for cataract, glaucoma, maculopathy, retinal detachment and other ocular disorders, and can thus result in substantial vision loss [1,2]. Recent studies indicated that early-onset myopia is becoming more common. For example, a study in Hong Kong showed that the prevalence of preschool myopia increased from 2.3% to 6.3% from 1996 to 1997 and from 2006 to 2007 [3]. Another two studies reported that the overall prevalence of myopia was 11.0% in Singaporean Chinese children aged 6 to 72 months [4] and 3.7% among preschoolers aged 3 to 6 years in Shanghai [5], respectively. Most importantly, the earlier the onset of myopia, the more likely high myopia will develop, and thus the worse the prognosis [6].

It has been well documented that both genetic and environmental factors are involved in the development of myopia. Generally, myopia has the trait of familial clustering [7], for example, one study of Singapore Chinese preschoolers showed that family history of myopia was the strongest risk factor for offsprings’ myopia [8]. Regarding environmental risk factors for myopia, prolonged near work, intensive education, and limited time spent outdoors are strongly supported [7]. For instance, Saw et. al, reported that young children with a greater reading exposure were more likely to be myopic [9]; a study in India showed that excessive screen time was associated with myopia in school-aged children [10], and another study of school-age children in northwest Ethiopia demonstrated that watching television from <2 m, and mobile exposure >4 h per day were significant risk factors for myopia [11].

With screen-based devices, e.g., tablets, smartphones, televisions, laptops and computers, more and more accessible nowadays, children are easily exposed to electronic screens at a very young age. It was reported that approximately 68% of children under age 3 use screen media on a daily basis [12]. Excessive screen time (2.8 h/day) exists in Shanghai preschool children [13]. In South Korea, most toddlers began using smart devices at 1–2 years old [14] and an increase was observed in the tendency of media device use among preschoolers [15]. Moreover, young Italian children were found overly exposed to mobile devices and most of them had their own device [16]; in America, a study showed that 96.6% young children aged 6 months to 4 years used mobile devices, and most started using before age 1 [17]. As mentioned above, screen exposure increased the risk of myopia in school children. Whether screen exposure in early life could impact the occurrence of myopia in preschool children remained unknown; therefore, this paper aimed to solve this issue.

## 2. Materials and Methods

### 2.1. Participants

Participants of this research were recruited from the baseline survey of the Longhua Child Cohort Study (LCCS) which mainly assessed the influence of early-life family and school environment upon psycho-behavioural development of Chinese children [18]. A total of 29,595 preschoolers aged 2 to 7 years participated in this study. Based on the questionnaire data, questionnaires were excluded if: (1) The child had a visual problem other than myopia; (2) there was missing information regarding screen exposure or eye conditions. The study was approved by the Ethics Committee of School of Public Health, Sun Yat-sen University in Guangzhou, China (No. 2015-016). Written informed consent was obtained from the guardians of all the preschoolers involved in the study. The study was carried out in accordance with the Declaration of Helsinki.

### 2.2. Data Collection

The primary caregiver was asked to complete a self-administered structured questionnaire regarding the child’s age, gender, premature birth (full term infant or premature infant), feeding patterns (breastfeeding, mixed feeding or bottle feeding), and the child’s previous screen exposure. Questions regarding parental age at childbirth, education level, monthly household income, and the refractive conditions of the parents (emmetropia, myopia or other visual disorders) were also included.

### 2.3. Measurement of Screen Exposure

For children aged no more than 6 years, screen exposure during the child’s whole life course was investigated; while for children aged over 6 years, only exposure from birth to 6 years was assessed.

The following questions were asked about the exposure: (1.1) Was the infant involved in watching television/laptop/computer screens when he/she was younger than 1 year of age? Two options: 0 = “no”, 1 = “yes”. (1.2) If your answer to question (1.1) was “yes”, how long on average per day was the infant exposed to the television screens? Five options: 1 = “<30 min”, 2 = “30–60 min”, 3 = “60–90 min”, 4 = “90–120 min”, 5 = “>2 h”. (2.1) Was the infant involved in using smartphones, tablets, or other handheld electronic screens when he/she was less than 1 year of age? Two options: 0 = “no”, 1 = “yes”. (2.2) If your answer to question (2.1) was “yes”, how long on average per day was the infant exposed to the electronic screens of those devices mentioned in (2.1)? Five options: 1 = “<30 min”, 2 = “30–60 min”, 3 = “60–90 min”, 4 = “90–120 min”, 5 = “>2 h”. Questions were repeated for yearly age bands up to 6 years of age. 

The answers to questions (1.2) and (2.2) were taken as the exposure score in the first year. If the answer to question (1.1) or (2.1) was “no”, the relevant device exposure score was recorded as zero. This was performed for similar questions across all age bands. The total exposure score, including exposure to screens of both the television and handheld electronic devices, was calculated by adding together the exposure scores across all age bands, and the average exposure score was obtained via dividing the total exposure score by the cumulative years of exposure. Finally, the average exposure score, ranging from 0 to 10, was converted into the average daily screen time based on the original exposure concerned questions. Namely, 0 meant no exposure; the score of 1 to 2, 2 to 4 and more than 4 meant the average daily screen time was less than 60, 60 to 120 and over 120 min, respectively.

### 2.4. Determination of the Presence of Myopia

Questions with regard to child’s eye conditions were listed as following [19]: (1) Has your child ever been diagnosed as having poor sight by the oculist? (0 = ‘no’, 1 = ‘yes’, 2 = ‘uncertain’); (2) and if yes, they were subsequently asked whether the child had been diagnosed as having astigmatism/myopia/hyperopia/strabismus/amblyopia/other common visual problems, respectively. In this study, a total of 26,433 children with no poor sight or those who were reported to be diagnosed as having only myopia were included in the analysis.

### 2.5. Covariates

According to previous literature [20,21], there has been a positive association of myopia with low birthweight for gestational age, gender, greater maternal age, and socioeconomic factors, and even a short period (1 month) of breastfeeding was protective against myopia. Thus the covariates for adjustment included children’s age, gender, premature birth, feeding patterns, parental age at childbirth, education level, and monthly household income. Moreover, the presence of parental myopia was the stratification variable in the stratified analysis.

### 2.6. Statistical Analyses

Covariates were compared using the Chi-square test or Fisher exact test, as appropriate, and presented as absolute frequencies and prevalence of myopia.

We tried to divide the electronic devices into the small handheld category and television/laptop/computer, and the results demonstrated in Tables S1 and S2 in the supplementary material suggested a similar impact. Thus we combined the exposure of the two kinds of electronic devices for further analyses.

Cox regression modelling [22,23] was adopted to generate prevalence ratio (PR) and 95% confidence intervals (95% CI) to examine the associations of the initial age of screen exposure and preschool myopia with adjustment for the aforementioned covariates. In the analysis, children were categorized into five subgroups in total, four subgroups based on the initial age (birth to 1, 1 to 2, 2 to 3 and after 3 years) of exposure and one subgroup that were never exposed.

Moreover, associations of average daily screen time with myopia in subgroups with different initial ages of exposure and associations between the total years of exposure with myopia in subgroups reporting different average daily screen time were explored via Cox regression modelling with adjustment for the aforementioned covariates.

With reference to the literature [24], we explored the sensitive/critical period for the impact of screen exposure on preschool myopia during the early life years (postnatal 3 years). A crossover analysis, based on different permutations of exposure (Yes) versus no exposure (No) in each year from birth to 3 years old, was performed via the Cox regression modelling with adjustment for the covariates.

The analyses were repeated for two subsets of preschoolers, one group whose parents reported they had good vision and the other where at least one parent was myopic. This was performed to determine the impact of screen exposure on myopia in children without a hereditary predisposition towards myopia and the combined impact of parental myopia and screen exposure on myopia.

SPSS version 23.0 (SPSS Inc., Chicago, IL) was employed to perform the statistical analysis, and the standard for statistical significance was set as two-tailed *p*-value < 0.05.

## 3. Results

### 3.1. Associations Between Children’s Characteristics and Preschool Myopia

Table 1 shows that the statistically significant differences in the prevalence of myopia based on premature birth, feeding patterns, gender, age of children, monthly household income, and parents’ visual status, but parents’ age at childbirth and education level were not significantly associated with myopia prevalence.

### 3.2. Associations Between the Initial Age of Screen Exposure and Preschool Myopia

**Figure 1:**
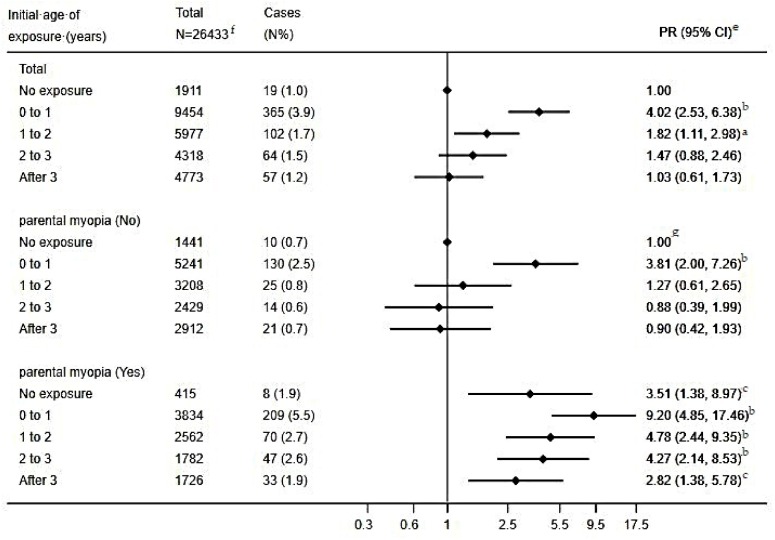
Association between the initial age of screen exposure and myopia amongst preschoolers. ^e^: Adjusted for children’s age, gender, feeding patterns, and premature birth; parental age at childbirth, education level, and monthly household income. ^f^: children with parents having other eyesight disorders were not included in the analysis. ^g^: This subgroup was the reference group for both children without/with parental myopia in the stratified analysis. ^a^: *p* < 0.05; ^b^: *p* < 0.001; ^c^: *p* < 0.01.

The association between the initial age of screen exposure and preschool myopia is shown in Figure 1. Analyses of the total participants presented that compared to preschoolers without screen exposure, those initially exposed during the first and second year of life had a statistically significant higher risk of myopia, and the adjusted PR (95% CI) were 4.02 (2.53, 6.38) and 1.82 (1.11, 2.98), respectively.

The reference group for the stratified analysis based on the presence of parental myopia was preschoolers without screen exposure and with parents having good eyesight. Among preschoolers whose parents had good eyesight, only those initially exposed during the first year of life had a statistically significant higher risk of myopia, and the adjusted PR (95% CI) was 3.81 (2.00, 7.26). For preschoolers with myopic parents but without screen exposure, the adjusted PR (95% CI) was 3.51 (1.38, 8.97); preschoolers with myopic parents and screen exposure all showed higher risk of myopia irrespective of the initial exposure age, and the adjusted PR (95% CI) decreased from 9.20 (4.85, 17.46) to 2.82 (1.38, 5.78) as the initial exposure age rose from 0–1 year to after 3 years.

### 3.3. Association Between Initial Age of Exposure, Average Daily Screen Time and Preschool Myopia

As is shown in Table 2, analyses of the total participants demonstrated that compared to children without screen exposure, those initially exposed to electronic screens during the first year of life all had significantly higher risk of myopia irrespective of the average daily screen time, while those initially exposed during age 1 to 2 showed higher risk of myopia when average daily screen time exceeded 60 min. Moreover, the risk of developing myopia was found to increase with exposure duration for children initially exposed to screens in the first and second year of life. When initial exposure occurred after age 2, a significantly higher risk of myopia was observed only in the subset of children with an initial age of exposure after age 3 and average daily screen time of over 120 min.

The reference group for the stratified analysis based on the presence of parental myopia was preschoolers without screen exposure and with parents having good eyesight. Among children without parental history of myopia, only those with initial screen exposure during the first year of life showed significantly higher risk of myopia, and longer average daily screen time was accompanied by a greater myopia risk, the adjusted PR (95% CI) varying from 2.83 (1.37, 5.85) to 6.54 (3.29, 13.03); moreover, the PR values for children with initial screen exposure after age 1 were all less than 2 and failed to show statistical significance. Among children with myopic parents but without screen exposure, the adjusted PR (95% CI) was 3.60 (1.41, 9.19). For children with myopic parents and screen exposure, significantly higher myopia risk was observed except for the subset of children who were initially exposed after 3 years with the daily duration of 60–120 min, and generally, myopia risk became higher when average daily screen time increased in each subgroup of children divided by initial age of screen exposure. The adjusted PR (95% CI) ranged from 7.37 (3.64, 14.90) to 10.74 (5.45, 21.14) for children with initial screen exposure during age 0–1; the adjusted PR (95% CI) ranged from 4.35 (2.07, 9.13) to 4.56 (1.96, 10.61) for children with initial screen exposure during age 1–2; the adjusted PR (95% CI) ranged from 2.81 (1.21, 6.54) to 3.36 (1.24, 9.13) for children with initial screen exposure during age 2–3; and the statistically significant adjusted PR (95% CI) values were 3.06 (1.30, 7.16) and 7.26 (2.73, 19.30) for children with initial screen exposure after age 3. More details are displayed in Table 2.

### 3.4. Association Between the Total Years of Exposure, Average Daily Screen Time and Preschool Myopia

Table 3 displays the association between the total years of exposure, average daily screen time and preschool myopia. Analyses of the total participants showed that compared to children never exposed to electronic devices, in the subgroup of daily average screen time less than 60 min, those exposed for 1 year, 4 years or more had a higher risk of myopia. When the daily average screen time varied from 60 to 120 min, those exposed for 4 years or more had a higher risk of myopia. In the group with daily average screen time more than 120 min, only those exposed for 3 years or more had a higher risk of myopia. 

In the stratified analysis based on the presence of parental myopia, the reference group was children never exposed to electronic devices and without myopic parents. In the subgroup of daily average screen time ranging from 60 to 120 min, children without myopic parents reporting no less than 5 years of exposure had a higher risk of myopia, the adjusted PR (95% CI) of which was 2.52 (1.24, 5.13); in the subgroup of daily average screen time over 120 min, children without myopic parents reporting 3 years or more exposure had a higher risk of myopia, and higher myopia risk was found as the total years of exposure increased, the adjusted PR (95% CI) varying from 2.88 (1.16, 7.17) to 5.80 (2.80, 12.01); while children with myopic parents showed higher risk of myopia irrespective of the daily average screen time and total years of screen exposure. For children with a history of parental myopia but without screen exposure, the adjusted PR (95% CI) was 3.48 (1.36, 8.89); for children with a history of parental myopia and with screen exposure, the adjusted PR was generally greater as the daily average screen time and total years of screen exposure increased. The adjusted PR (95% CI) ranged from 3.41 (1.52, 7.66) to 8.88 (4.11, 19.17) for children with daily average screen time of less than 60 min; the adjusted PR (95% CI) ranged from 2.62 (1.05, 6.57) to 8.26 (4.21, 16.18) for children with daily average screen time of 60–120 min; and the adjusted PR (95% CI) ranged from 6.41 (2.12, 19.37) to 9.41 (4.60, 19.25) for children with daily average screen time of more than 120 min. More details can be seen in Table 3.

### 3.5. Associations Between Screen Exposure During the Early Stage of Life (Postnatal Three Years) and Preschool Myopia

A crossover analysis, based on exposure (yes) versus no exposure (no) during each year from birth to 3 years old, was performed to explore the sensitive/critical period for the impact of screen exposure on preschool myopia during the early life years (Table 4). Analyses of the total participants showed that compared to children without screen exposure during the postnatal three years, all the subgroups exposed during the first year had a statistically significant higher risk of myopia than those not exposed during the first year, the adjusted PR (95% CI) varying from 2.91 (1.76, 4.82) to 4.33 (2.71, 6.91), and children exposed only during the postnatal second year did not show a higher risk of myopia.

In the stratified analysis based on the presence of parental myopia, the reference group was children never exposed to electronic devices during the postnatal three years and without myopic parents. Among children without parental history of myopia, only those subsets of children who were exposed (1) merely in the first year or (2) in the combination of first year with second and/or third year showed higher risk of myopia, and the adjusted PR (95% CI) ranged from 3.52 (1.76, 7.01) to 7.03 (3.73, 13.26). Among children with myopic parents but without screen exposure, the adjusted PR (95% CI) was 3.06 (1.90, 4.91). And among children with myopic parents and screen exposure, all groups showed a higher risk of myopia except for the subset of children exposed only in the postnatal second year, and the statistically significant adjusted PR (95% CI) varied from 4.46 (2.80, 7.08) to 11.35 (6.26, 20.60). More details can be found in Table 4.

## 4. Discussion

Studies concerning screen exposure and myopia have been conducted worldwide such as in China, Japan, India, Ireland and Denmark with a focus on school-age children, which led to discrepant findings. A study involving primary and middle school students in six provinces of China showed children had a higher risk of myopia whose parents did not limit their offspring’s screen time [25]; Siofra and colleagues reported that using screens >3 h per day was associated with a higher risk of myopia among schoolchildren in Ireland [26]; the North India Myopia Study found screen viewing was a significant risk factor for myopia progression amongst children aged 5 to 15 years [10]; and the Copenhagen Child Cohort 2000 Eye Study revealed that using screen devices >6 h/day, compared to screen device use <2 h/day, induced a roughly doubled risk of having myopia among 16–17 year-old adolescents [27]. Moreover, another two studies in older children aged 6 to 18 years in Qatar demonstrated a highly positive association between prolonged screen time (more than 3 h/day) and poor vision [28,29]. However, Hiroto et al. found use of screen devices was not significantly independently correlated with axial length which is longer in myopic eyes in a sample of 122 Japanese third grade students [30], and a study in Taiwan reported use of computer, Internet, and games (0.68 ± 0.86 h/day on average) is not related to incident myopia among 7–12 year-old children [31]. The difference in the findings might lie in the sample size and the assessment of screen exposure and myopia.

While the present study was focused on screen exposure and myopia among preschoolers, and provided more details regarding the relationship between screen exposure and myopia. Results of the study suggested the hypothesis that screen exposure in early life could be associated with a higher risk of preschool myopia, in favour of this hypothesis was the risk of having myopia increasing with the daily exposure duration and total years of exposure, especially during the early life years. Although there were no other studies of large groups of preschoolers available to directly compare our data to, our study reinforced, to some extent, the finding of the majority of the previous researches that the screen exposure was a risk factor for myopia.

When exposure during a certain period has a stronger impact on development and subsequent disease risk than it would at other times, this period may be regarded as the sensitive period for this exposure having the certain impact [24]. Intriguingly, for children whose parents had no poor sight, the cross-over analysis (Table 4) showed that screen exposure in the postnatal first year only or in the combination of the postnatal first year with the second year or/and the third year translated into a much higher risk of preschool myopia, while screen exposure in the postnatal second year or/and the third year showed insignificant association with preschool myopia. Moreover, as is shown in Figure 1, for children without myopic parents, only those initially exposed to electronic devices during the postnatal first year showed a higher risk of preschool myopia. Therefore, it was hypothesized that the postnatal first year might be the sensitive period in early life for the association between screen exposure and preschool myopia. This finding might be explained by the fact that basic visual function develops soon after birth and vision improves rapidly during the early infancy [32,33,34,35,36], and that the growth of the eye is greatest during this period and slows with age [37,38]. To the best of our knowledge, our study was the first to explore the sensitive period for the impact of screen exposure on myopia. Additionally, the American Academy of Pediatrics recommended that children under 18 months should avoid screen exposure [39], and both the Italian and the Canadian Pediatric Society have made the guideline that screen time for children younger than 2 years is not advocated [16,40]. Our finding, if adequately confirmed in further studies, could help to strengthen and improve these recommendations.

In the past decades, several studies consistently reported the strong positive association between parental myopia and children’s risk of myopia. For example, a meta-analysis summarized that the odds ratio (OR) of giving birth to a myopic child was 2.10 when one parent had myopia and 2.13 when two parents had myopia [41]. A study in Singapore showed that children with two myopic parents were more likely to be myopic than children without myopic parents [42]. A 22-year follow-up study in Finland found higher myopic progression among females with myopic parents than those with non-myopic parents [43]. In line with previous findings, our study also found that among preschoolers never exposed to electronic screens, children with one or two myopic parents had a significantly higher risk of myopia than those with parents with good eyesight. Thus, all these studies suggest that parental myopic status is an important risk factor for offsprings’ myopia.

Regarding the mechanism of the hypothesis that screen exposure could be associated with a higher risk of preschool myopia, we propose the following which hopefully facilitates understanding of our findings. First, previous studies of older children or adults demonstrated focusing errors increase the closer the viewing distance [44,45] and high lags of accommodation are associated with myopia [44,46]. However, according to our data, there was no significant difference between the impact of viewing hand-held devices and television/computer/laptop screens, and infants naturally manipulate objects at a very short distance, thus, the performance of near tasks by itself cannot explain the findings. Normally, attention and focus will frequently shift from near objects to far and back, whereas what is displayed on the screen devices, e.g., video games, tend to attract prolonged attention that would usually not occur until children learn to read. Moreover, the human eye generally undergoes a rapid growth from birth to 3 years of age and the variability of refractive error decreases progressively during this period [47], and the greatest increase in eye growth, which is measured by axial length, occurs during the first 10 months of life [48]. Therefore it is speculated that the steady fixation at a specific distance might contribute to myopia for very young children’s eyes, and one reason that outdoor activities appear to be beneficial [49,50,51,52] might be that they encourage more frequent shifts of attention and focus. If sufficiently confirmed in further studies, the speculation could provide insight into the mechanism of myopia. Second, a series of studies consistently showed that children who spent more time outdoors were significantly and negatively associated with incident myopia [49,50,51,52], and low illuminance levels indoors can cause myopia in animal models [53]. More time spent indoors watching electronic screens could translate into less time spent outdoors and insufficient exposure to natural light, and thus increase the risk of preschool myopia. Third, in infants, the macular pigment density is low [54], and the fovea not well developed [55], thus, the defocus detection system is not really ready for light exposure from unnatural sources. After all, this correlational study is incapable of justifying the aforementioned possible mechanisms. Future research is warranted to elaborate the underlying mechanisms further.

The findings need to be interpreted in conjunction with the following limitations. First, the data were obtained retrospectively by means of self-report, thus, recall bias would be inevitable. Second, diagnosed myopia in preschool children is relatively rare, and the fact that a child’s vision has been examined professionally may be influenced by parental characteristics such as myopia and education, thus opening up the possibility of extensive confounding, which may also affect the estimates of screen time, and might further distort the true association between screen exposure and preschool myopia. In the future research, cycloplegic refraction [56] with the appropriate cycloplegic regime, which is recognized as the gold standard for epidemiological studies, or the axial length (AL)/corneal radius (CR) ratio as the best surrogate measure, is recommended to assess the outcome directly; better methods to measure screen exposure, such as keeping a diary of screen time, would yield more convincing exposure data. Third, the measurement of outcome was monotonous and not graded (report of a diagnosis of myopia). It would be preferred to measure children’s refractive error, corneal curvature, lens thickness, axial length and other parameters for further elucidating the impact of screen exposure on preschool visual function; information about screen exposure when caregivers were separated from children, e.g., when children were in kindergarten, might not be fully reported, which could lead to an underestimation of screen exposure. Fourth, older children may do a fair amount of near viewing for things other than screens, which was not measured in this study. A complete record of near work in future research would facilitate the assessment of how close viewing, in general, may contribute to the increased rate of myopia in children.

## 5. Conclusions

Our findings suggested the hypothesis that screen exposure in early life could be associated with a higher risk of preschool myopia and the postnatal first year might be the sensitive period for the association. However, considering the poor assessment of myopia in our study, it is premature to conclude that early screen time leads to myopia with current data. More longitudinal research performed with cycloplegia would be essential to establish the causal link between screen exposure in early life and myopia; a more important question is whether early screen exposure contributes to the later myopia epidemic of school-age children. Further longitudinal studies employing cycloplegia would then be required to shed light upon this issue.

## Figures and Tables

**Table 1:** Relationships between the reported characteristics of children and the presence of myopia.

Characteristics	No. of Children	No. with Myopia	Myopia Prevalence (%)	*χ* ^2^
*Gender*				4.18 ^a^
Male	14,335	354	2.5	
Female	12,098	253	2.1	
*Age (years)*				103.52 ^b^
<3	524	3	0.6	
3 to 4	7254	76	1.0	
4 to 5	9010	207	2.3	
5 to 6	8793	290	3.3	
≥6	852	31	3.6	
*Premature birth*				16.56 ^b^
No	24,472	536	2.2	
Yes	1961	71	3.6	
*Feeding patterns*				25.48 ^b^
Breastfeeding	5557	101	1.8	
Mixed feeding	18,296	413	2.3	
Bottle feeding	2580	93	3.6	
*Maternal age at childbirth(years)*				5.86
<20	941	24	2.6	
20 to 30	20,338	451	2.2	
30 to 40	5009	125	2.5	
>40	145	7	4.8	
*Paternal age at childbirth(years)*				2.06
<20	311	9	2.9	
20 to 30	16,345	374	2.3	
30 to 40	9031	202	2.2	
>40	746	22	2.9	
*Maternal education level*				3.80
<Undergraduate	11,368	284	2.5	
Undergraduate	14,526	310	2.1	
>Undergraduate	539	13	2.4	
*Paternal education level*				*3.92*
<Undergraduate	10,340	255	2.5	
Undergraduate	15,130	325	2.1	
>Undergraduate	963	27	2.8	
*Monthly household income (￥)*				10.11 ^a^
<5000	3954	106	2.7	
5000 to 10,000	6929	174	2.5	
10,000 to 15,000	5048	112	2.2	
15,000 to 25,000	5748	131	2.3	
>25,000	4754	84	1.8	
*Paternal visual status*				91.10 ^b^
Emmetropia	19,097	337	1.8	
Myopia	6817	244	3.6	
Other visual disorders	519	26	5.0	
*Maternal visual status*				120.45 ^b^
Emmetropia	19,101	319	1.7	
Myopia	6942	272	3.9	
Other visual disorders	390	16	4.1	

^a^: *p* < 0.05; ^b^: *p* < 0.001.

**Table 2:** Association between initial age of exposure, average daily screen time and preschool myopia ^e^.

Initial Age of Exposure (Years)	Average Daily Screen Time (Minutes)	Total (*N* = 26,433)			Presence of Parental Myopia (*N* = 25,550) ^f^					
					No (*N* = 15,231)			Yes (*N* = 10,319)		
		No. of Children	Cases (*n* %)	PR (95% CI)	No. of Children	Cases (*n* %)	PR (95% CI)	No. of Children	Cases (*n* %)	PR (95% CI)
	0	1911	19 (1.0)	1.00	1441	10 (0.7)	1.00 ^g^	415	8 (1.9)	3.60 (1.41, 9.19) ^b^
0 to 1										
	<60	2576	72 (2.8)	2.99 (1.80, 4.96) ^b^	1540	28 (1.8)	2.83 (1.37, 5.85) ^c^	936	39 (4.2)	7.37 (3.64, 14.90) ^b^
	60–120	4842	179 (3.7)	3.74 (2.32, 6.00) ^b^	2645	56 (2.1)	3.09 (1.57, 6.07) ^c^	1995	107 (5.4)	8.75 (4.53, 16.90) ^b^
	>120	2036	114 (5.6)	5.62 (3.45, 9.16) ^b^	1056	46 (4.4)	6.54 (3.29, 13.03) ^b^	903	63 (7.0)	10.74 (5.45, 21.14) ^b^
1 to 2										
	<60	1833	28 (1.5)	1.72 (0.95, 3.11)	1000	5 (0.5)	0.89 (0.30, 2.65)	769	21 (2.7)	4.62 (2.08, 10.26) ^b^
	60–120	3043	49 (1.6)	1.72 (1.01, 2.95) ^a^	1647	13 (0.8)	1.25 (0.54, 2.91)	1301	34 (2.6)	4.35 (2.07, 9.13) ^b^
	>120	1101	25 (2.3)	2.29 (1.24, 4.22) ^c^	561	7 (1.2)	1.94 (0.73, 5.20)	492	15 (3.0)	4.56 (1.96, 10.61) ^b^
2 to 3										
	<60	1623	21 (1.3)	1.29 (0.69, 2.42)	927	6 (0.6)	1.01 (0.36, 2.83)	671	15 (2.2)	2.81 (1.21, 6.54) ^a^
	60–120	2091	31 (1.5)	1.26 (0.70, 2.26)	1166	4 (0.3)	0.46 (0.14, 1.48)	863	25 (2.9)	3.15 (1.45, 6.87) ^c^
	>120	604	12 (2.0)	1.74 (0.84, 3.62)	336	4 (1.2)	1.70 (0.52, 5.57)	248	7 (2.8)	3.36 (1.24, 9.13) ^a^
After 3										
	<60	2332	27 (1.2)	1.06 (0.59, 1.93)	1433	9 (0.6)	0.80 (0.32, 1.99)	834	16 (1.9)	3.06 (1.30, 7.16) ^a^
	60–120	1911	17 (0.9)	0.78 (0.40, 1.53)	1156	7 (0.6)	0.68 (0.26, 1.83)	706	9 (1.3)	2.08 (0.80, 5.42)
	>120	530	13 (2.5)	2.08 (1.01, 4.29) ^a^	323	5 (1.5)	1.68 (0.55, 5.09)	186	8 (4.3)	7.26 (2.73, 19.30) ^b^

^e^: adjusted for children’s age, gender, feeding patterns, and premature birth; parental age at childbirth, education level, and monthly household income. ^f^: children with parents having other eyesight disorders were not included in the analysis. ^g^: This subgroup was the reference group for both children without/with parental myopia in the stratified analysis; ^a^: *p* < 0.05; ^b^: *p* < 0.001; ^c^: *p* < 0.01.

**Table 3:** Association between the total years of exposure, average daily screen time and preschool myopia ^e^.

Daily Average Screen Time	Total Years of Screen Exposure	Total (*N* = 26,433)			Presence of Parental Myopia (*N* = 25,550) ^f^					
					No (*N* = 15,231)			Yes (*N* = 10,319)		
		No. of Children	Cases (*n* %)	PR (95% CI)	No. of Children	Cases (*n* %)	PR (95% CI)	No. of Children	Cases (*n* %)	PR (95% CI)
0	0	1911	19 (1.0)	1.00	1441	10 (0.7)	1.00 ^g^	415	8 (1.9)	3.48 (1.36, 8.89) ^b^
<60 min										
	1	1423	24 (1.7)	2.01 (1.09, 3.70) ^a^	932	6 (0.6)	1.15 (0.41, 3.19)	465	16 (3.5)	6.99 (3.06, 15.93) ^b^
	2	1969	25 (1.3)	1.42 (0.78, 2.60)	1187	8 (0.7)	1.14 (0.44, 2.91)	726	17 (2.3)	4.35 (1.93, 9.83) ^b^
	3	2260	34 (1.5)	1.58 (0.90, 2.78)	1273	13 (1.0)	1.55 (0.68, 3.55)	911	17 (1.9)	3.41 (1.52, 7.66) ^c^
	4	1577	29 (1.8)	1.90 (1.06, 3.41) ^a^	854	12 (1.4)	2.14 (0.92, 4.98)	669	16 (2.4)	4.34 (1.92, 9.81) ^b^
	≥5	1135	36 (3.2)	2.76 (1.56, 4.86) ^b^	654	9 (1.4)	1.65 (0.66, 4.12)	448	25 (5.6)	8.88 (4.11, 19.17) ^b^
60–120 min										
	1	483	2 (0.4)	0.46 (0.11, 2.00)	282	1 (0.4)	0.56 (0.07, 4.40)	184	0 (0.0)	---
	2	1582	16 (1.0)	1.09 (0.56, 2.14)	928	6 (0.6)	1.07 (0.38, 2.99)	608	9 (1.5)	2.62 (1.05, 6.57) ^a^
	3	2981	40 (1.3)	1.33 (0.76, 2.30)	1689	11 (0.7)	0.96 (0.40, 2.26)	1220	28 (2.3)	3.59 (1.71, 7.55) ^c^
	4	3294	74 (2.2)	2.24 (1.35, 3.72) ^c^	1793	21 (1.2)	1.71 (0.80, 3.65)	1370	47 (3.4)	5.20 (2.59, 10.45) ^b^
	≥5	3547	144 (4.1)	3.42 (2.09, 5.57) ^b^	1922	41 (2.1)	2.52 (1.24, 5.13) ^a^	1483	91 (6.1)	8.26 (4.21, 16.18) ^b^
>120 min										
	1	126	1 (0.8)	0.91 (0.18, 4.59)	69	1 (1.4)	2.94 (0.37, 23.36)	52	0 (0.0)	---
	2	367	7 (1.9)	1.46 (0.63, 3.39)	216	2 (0.9)	1.45 (0.31, 6.67)	138	5 (3.6)	6.41 (2.12, 19.37) ^c^
	3	941	26 (2.8)	2.01 (1.17, 3.44) ^a^	528	9 (1.7)	2.88 (1.16, 7.17) ^a^	382	16 (4.2)	6.79 (3.01, 15.33) ^b^
	4	1203	38 (3.2)	2.28 (1.36, 3.83) ^c^	617	12 (1.9)	3.19 (1.36, 7.51) ^c^	528	24 (4.5)	6.72 (3.12, 14.44) ^b^
	≥5	1634	92 (5.6)	3.68 (2.32, 5.82) ^b^	846	38 (4.5)	5.80 (2.80, 12.01) ^b^	729	48 (6.6)	9.41 (4.60, 19.25) ^b^

^e^: adjusted for children’s age, gender, feeding patterns, and premature birth; parental age at childbirth, education level, and monthly household income. ^f^: Children with parents having other eyesight disorders were not included in the analysis. ^a^: *p* < 0.05; ^b^: *p* < 0.001; ^c^: *p* < 0.01. ---: Coefficients did not converge. ^g^: This subgroup was the reference group for both children without/with parental myopia in the stratified analysis.

**Table 4:** Associations between screen exposure during the early stage of life (postnatal three years) and preschool myopia ^e^.

Age-Specific Exposure (Years)			Total (*N* = 26,433)			Presence of Parental Myopia (*N* = 25,550) ^f^					
						No (*N* = 15,231)			Yes (*N* = 10,319)		
0–1	1–2	2–3	No. of Children	Cases (*n* %)	PR (95% CI)	No. of Children	Cases (*n* %)	PR (95% CI)	No. of Children	Cases (*n* %)	PR (95% CI)
No	No	No	6684	76 (1.1)	1.00	4353	31 (0.7)	1.00 ^g^	2141	41 (1.9)	3.06 (1.90, 4.91) ^b^
Yes	No	No	726	29 (4.0)	3.67 (2.39, 5.63) ^b^	453	11 (2.4)	3.52 (1.76, 7.01) ^b^	244	17 (7.0)	11.35 (6.26, 20.60) ^b^
No	Yes	No	454	5 (1.1)	1.04 (0.42, 2.58)	288	3 (1.0)	1.62 (0.49, 5.29)	149	2 (1.3)	2.08 (0.50, 8.71)
No	No	Yes	4318	64 (1.5)	1.44 (1.03, 2.01) ^a^	2429	14 (0.6)	0.94 (0.50, 1.78)	1782	47 (2.6)	4.46 (2.80, 7.08) ^b^
Yes	Yes	No	512	23 (4.5)	4.33 (2.71, 6.91) ^b^	313	14 (4.5)	7.03 (3.73, 13.26) ^b^	179	9 (5.0)	7.99 (3.79, 16.82) ^b^
No	Yes	Yes	5523	97 (1.8)	1.86 (1.37, 2.51) ^b^	2920	22 (0.8)	1.33 (0.77, 2.30)	2413	68 (2.8)	5.22 (3.38, 8.06) ^b^
Yes	No	Yes	619	19 (3.1)	2.91 (1.76, 4.82) ^b^	382	10 (2.6)	3.98 (1.95, 8.14) ^b^	210	6 (2.9)	4.64 (1.93, 11.16) ^c^
Yes	Yes	Yes	7597	294 (3.9)	4.04 (3.13, 5.21) ^b^	4093	95 (2.3)	3.91 (2.60, 5.90) ^b^	3201	177 (5.5)	9.92 (6.74, 14.62) ^b^

^e^: adjusted for children’s age, gender, feeding patterns, and premature birth; parental age at childbirth, education level, and monthly household income. ^f^: children with parents having other eyesight disorders were not included in the analysis. ^g^: This subgroup was the reference group for both children without/with parental myopia in the stratified analysis. ^a^: *p* < 0.05; ^b^: *p* < 0.001; ^c^: *p* < 0.01.

## Supplementary Materials

The following are available online at https://www.mdpi.com/1660-4601/17/3/1056/s1, Table S1: Association between initial age of exposure to handheld electronic devices and myopia, Table S2: Association between the initial age of television/laptop/computer screen exposure and myopia.
