# Supplementary material for: Associations Between Screen Exposure in Early Life and Myopia amongst Chinese Preschoolers
Source: Int J Environ Res Public Health. 2020 Feb 7;17(3):1056. doi: 10.3390/ijerph17031056 (PMC7037286; doi:10.3390/ijerph17031056)
Supplement: Supplementary file 1 [file ijerph-17-01056-s001.pdf]

International Journal of
Environmental Research and Public Health

Supplementary Material:

Table S1. Association between initial age of exposure to handheld electronic devices and myopia.

Initial Age of Exposure (Years)	No. Of Children	No. of Case	Myopia Prevalence (%)	PR (95% CI)	Adjusted PR [a] (95% CI)	Adjusted PR [b] (95% CI)
No exposure	1911	19	1.0	1.00	1.00	1.00
0 to 1	4916	221	4.5	4.91 (3.07, 7.84) ***	4.91 (3.07, 7.85) ***	4.41 (2.19, 8.90) ***
1 to 2	4683	99	2.1	2.38 (1.46, 3.89) **	2.38 (1.45, 3.89) **	2.46 (1.20, 5.06) *
2 to 3	4805	84	1.7	1.83 (1.11, 3.02) *	1.84 (1.12, 3.03) *	2.02 (0.97, 4.17)
After 3	5978	104	1.7	1.36 (0.84, 2.22)	1.41 (0.86, 2.31)	1.78 (0.87, 3.65)

[a]: Adjusted for children's age, gender, feeding patterns, and premature birth; parental age at childbirth, education level, visual conditions and monthly household income. [b]: Adjusted for a + exposure to television/laptop/computer; *: p <0.05; **: p < 0.01; ***: p < 0.001.

Table S2. Association between the initial age of television/laptop/computer screen exposure and myopia.

Initial Age of Exposure (Years)	No. Of Children	No. of Case	Myopia Prevalence (%)	PR (95% CI)	Adjusted PR [a] (95% CI)	Adjusted PR [b] (95% CI)
No exposure	1911	19	1.0	1.00	1.00	1.00
0 to 1	7291	300	4.1	4.15 (2.61, 6.60) ***	4.20 (2.64, 6.69) ***	3.34 (2.00, 5.56) ***
1 to 2	5486	116	2.1	2.20 (1.35, 3.57) **	2.22 (1.36, 3.61) **	2.02 (1.19, 3.43) *
2 to 3	3868	69	1.8	1.74 (1.05, 2.90) *	1.78 (1.07, 2.95) *	1.82 (1.05, 3.15) *
After 3	7141	89	2.3	1.11 (0.68, 1.82)	1.13 (0.69, 1.85)	1.17 (0.69, 1.98)

[a]: Adjusted for children's age, gender, feeding patterns, and premature birth; parental age at childbirth, education level, visual conditions and monthly household income. [b]: Adjusted for a + exposure to handheld electronic devices; *: p <0.05; **: p < 0.01; ***: p < 0.001.
